# Caloric restriction or telmisartan control dyslipidemia and nephropathy in obese diabetic Zücker rats

**DOI:** 10.1186/1758-5996-6-10

**Published:** 2014-01-27

**Authors:** Eduardo J Lezcano, Pablo Iñigo, Ana M Larraga, Cristina Barranquero, Ignacio Gimenez, Jesús Osada

**Affiliations:** 1 Servicio de Cardiología, Hospital de San Pedro, Logroño, Calle Piqueras, 98 26006 Logroño, La Rioja, Spain; 2 Departamento de Medicina. Facultad de Medicina. Servicio de Nefrología, Hospital Clínico Universitario “Lozano Blesa”, Universidad de Zaragoza, Zaragoza, Spain; 3 Departamento de Farmacología y Fisiología, Universidad de Zaragoza, Zaragoza, Spain; 4 Departamento de Bioquímica y Biología Molecular y Celular, Facultad de Veterinaria, Instituto de Investigación Sanitaria de Aragón - Universidad de Zaragoza, Zaragoza, Spain; 5 CIBER de Fisiopatología de la Obesidad y Nutrición, Instituto de Salud Carlos III, Madrid, Spain

**Keywords:** Zücker diabetic fatty rat, Type II diabetes, Dyslipidaemia, Caloric restriction, Telmisartan, Mildly oxidized LDL

## Abstract

**Background:**

The obese Zücker diabetic fatty male rat (ZDF:Gmi™-fa) is an animal model of type II diabetes associated with obesity and related metabolic disturbances like dyslipidaemia and diabetic nephropathy. In addition, diabetic dyslipidaemia has been linked to vascular and glomerular damage too. Dietary fat restriction is a current strategy to tackle obesity and, telmisartan, as a renoprotective agent, may mediate cholesterol efflux by activating PPARγ. To test the hypothesis that both therapeutical alternatives may influence dyslipidaemia and nephropathy in the ZDF rat, we studied their effect on development of diabetes.

**Methods:**

Male Zücker Diabetic Fatty (ZDF) rats received a low-calorie diet, vehicle or telmisartan for 9 weeks. Blood samples were obtained for analyses of lipids and lipoproteins, LDL-oxidisability, HDL structural and functional properties. Urinalysis was carried out to estimate albumin loss. At the end of the experimental period, rats were sacrificed, liver extracted and *APOA1* mRNA quantified.

**Results:**

Results indicated that low-calorie diet and telmisartan can slower the onset of overt hyperglycaemia and renal damage assessed as albuminuria. Both interventions decreased the oxidative susceptibility of LDL and hepatic *APOA1* mRNA expression but only dietary restriction lowered hyperlipidaemia.

**Conclusion:**

Either a dietary or pharmacologic interventions with telmisartan have important beneficial effects in terms of LDL oxidative susceptibility and progression of albuminuria in obesity related type II diabetes.

## Background

Incidence and prevalence of obesity and diabetes have increased considerably in recent years [1,2]. They both represent a major public health concern in terms of morbidity and mortality, due to their association with cardiovascular disorders, resulting from metabolic disturbances (atherogenic dyslipidaemia and hyperglycaemia), inflammatory status and endothelial dysfunction that led to atherosclerosis [3,4].

Zücker obese and diabetic rat (ZDF/Gmi-fa) is an animal model frequently used to study obesity and related metabolic disturbances like dyslipidaemia and non-insulin dependent diabetes [5]. They also develop diabetic nephropathy with proteinuria and focal glomerulosclerosis that end in renal insufficiency [6].

Low serum levels of HDL cholesterol (HDL-c) are consistently associated with increased risk for all forms of atherosclerotic disease and its clinical sequelae, including myocardial infarction, stroke, and sudden death. HDL particles have been studied in vitro, in animal models, and in humans. The classical function of HDL is reverse cholesterol transport from macrophages. However, HDL particles inhibit many inflammatory activities, inhibit oxidation of lipoproteins and cell membranes, and help to maintain endothelial function and integrity too. They also exert anticoagulant effects by inhibiting coagulation proteins and platelet aggregation. Furthermore, HDL has been found to improve beta cell survival and insulin secretion in diabetic patients and to reduce insulin resistance in muscle and adipose tissues [7]. HDL particles are very heterogeneous; they differ in shape and size. APOA1 plays a key role for cholesterol efflux, but there are other components of the HDL particle such as apolipoproteins, antioxidant enzymes or lipids besides cholesterol that confer HDL its beneficial properties [8]. In patients with certain diseases (diabetes, cardiovascular diseases, etc.), the different proteins and lipids commonly found in HDL particles may undergo modifications such as amino acid oxidation, protein loss, proteolytic degradation, protein glycation, and many other harmful modifications that interfere with HDL function [9,10].

There is a beneficial effect of dietary restriction and weight reduction on the hyperglycaemia of patients with obesity-associated non-insulin-dependent diabetes mellitus (NIDDM). A low-energy diet recommended for the treatment of obesity should be low fat (25-35% total energy), high carbohydrate, high protein, and high fiber [11,12]. Caloric restriction in obese pre-diabetic rats prevents hyperglycaemia, beta-cell depletion, loss of beta-cell GLUT 2 and glucose incompetence [21].

Nuclear PPARγ receptor is a transcription factor regulating many genes related to adipogenesis, lipid metabolism and insulin sensitivity [13]. Mainly known for its angiotensin II receptor blocker (ARB) and anti-hypertensive action, telmisartan is also a partial PPARγ agonist [14,15]. The purpose of this study was to examine the effects of PPARγ modulation using telmisartan, or caloric restriction on plasma lipoprotein profile, plasma APOA1 and its hepatic expression, in an early stage of vascular disease (with or without albuminuria) in obese and diabetic Zücker rats.

## Methods

### Animals

Male Zücker obese (fa/fa) and Male Zücker Diabetic Fatty (ZDF) rats, weighing 250–300 g (purchased at 8 weeks of age from Charles River, Barcelona, Spain), were used for experiments. Rats, housed in sterile filter-top cages, were acclimatized in a room maintained at 20°C with a 12-h light–dark cycle for 10 days, allowed ad libitum access to water and standard chow diet (Purina LabDiet^®^ 5008 diet; Purina S.A. Spain). ZDF rats were randomly assigned to three groups of eight animals each: a low-calorie diet (ZDFD), vehicle (ZDFV) or treatment (1.5 mg/kg telmisartan, ZDFT). In addition, male Zücker rats (n = 5) were used as control to observe the early effects of obesity related diabetes. Animals in the hypocaloric diet were fed with a 30% low-calorie diet (Purina LabDiet^®^ 5008 diet)*.* Telmisartan (Boehringer Ingelheim Pharma KG, Ingelheim, Germany) was dissolved in 1 N NaOH and then in water, and the solution adjusted at pH 7.4. Telmisartan (1.5 mg/kg/day) or the vehicle were orally administered once daily in the morning from weeks 10 to 19. The animals were handled and killed always observing criteria from the European Union for care and use of laboratory animals in research, and the protocol was approved by the Ethics Committee for Animal Research of the University of Zaragoza.

Blood samples were obtained from tail vein at weeks 10 (after 2-week quarantine), 14, and 19, after an 18 h-fasting period. Blood samples were collected in heparin coated capillary tubes and centrifuged at 2000 g for 5 min. At the moment of sacrifice (18 h after the feeding), rats were anesthetized with 1 ml of 8% Avertine (Aldrich Chemical Co., Madrid, Spain) in 0.1 M phosphate, pH 7.2, and blood drawn from hearts. Blood was collected in tubes containing 1 g/l sodium EDTA. Liver was removed and quickly frozen in liquid N_2_ until total RNA was extracted.

### Urinalysis

Twenty-four-hour urine samples were collected from all animals using metabolic cages while providing food and water. After urine volume was measured, the samples were centrifuged at 1500 rpm for 5 minutes at room temperature in order to separate the supernatant from the residue. Urinary albumin was measured with an immunoenzymatic assay method Rat Albumin EIA kit (SPI bio, France).

### Blood chemistry

In all animals, plasma creatinine was assayed using QuantiChrom™ Creatinine Assay Kit (*DICT-500) (*BioAssay Hayward*, CA, USA*), blood urea nitrogen (BUN) and glucose were determined using Vetscan (Abaxis, Inc., USA), analyzer.

### Lipid and lipoprotein analyses

Total plasma cholesterol, triglyceride (corrected for free glycerol) and non-esterified fatty acids (NEFA) concentrations were quantified enzymatically in a microtiter assay using commercial kits (Thermo scientific, Madrid, Spain) and (Wako, Madrid, Spain). Cardiolipid (Sigma) was used as quality control. Plasma lipoprotein profile was determined in 100 μl of plasma samples by fast protein liquid chromatography (FPLC) gel filtration using a Superose 6B column (Amersham Pharmacia, Barcelona, Spain), and the total cholesterol in each fraction was measured using a fluorometric method (Amplex Red, Molecular Probes, USA). Apolipoprotein, APOA1, was quantified by ELISA using specific polyclonal antibodies (Biodesign, Saco, ME, USA), as previously described [16].

### LDL oxidation susceptibility

LDL oxidisability was assessed using modified procedures of Navab et al. [17] to determine the presence of reactive oxygen species (ROS) by measuring the conversion of 2’,7’-dichlorofluorescein diacetate (DCFH-DA) into fluorescent dichlorofluorescein (DCF). Briefly, the FPLC-separated LDL fractions (5 μg of cholesterol) were incubated, at 37°C with 2 microg of DCFH-DA, in 25 μl of 0.1% sodium azide and 100 μl of PBS, up to a total volume of 150 μl. The fluorescence was measured after 3 h of incubation at 485 nm excitation and 535 nm emission wavelengths. The ability of HDL to inactivate LDL ROS was also tested.

### RNA isolation

RNA from each liver was isolated using Tri reagent (Sigma). DNA contaminants were removed by TURBO DNAse treatment using the DNA removal kit from AMBION (Austin, TX, USA). RNA was quantified by absorbance at 260 and 280 nm. The A_260_/_280_ ratio did not vary significantly among groups. The integrity of the 28S and 18S ribosomal RNAs was verified by 1% agarose gel electrophoresis of 500 ng of total RNA. Ethidium-bromide stained gels were exposed to UV light and images were captured (BioRad, Madrid, SpA-In). Intensity of bands for each condition was calculated using Quantity One^®^ software version 4.5.0 (BioRad). The 28S/18S ratio did not differ among groups. RNA integrity number (RIN) from samples was obtained by RNA nano kit using an Agilent 2100 Bioanalyzer. RIN values were not significantly different among tested groups.

### Quantification of mRNA

The mRNA expression was analysed by reverse transcriptase and quantitative real-time polymerase chain reaction (RT-qPCR). Equal amounts of DNA-free RNA from each sample of each animal were used. First-strand cDNA synthesis and the PCRs were performed using the SuperScript II Platinum Two-Step RT-qPCR Kit with SYBR Green (Invitrogen, Madrid, Spain), according to the manufacturer’s instructions and as previously described [18]. Primers were designed by Primer Express^®^ (Applied Biosystems, Foster City, CA) and checked by BLAST analysis (NCBI) to verify specificity and selective amplification of the target gene as well as to get amplification of the cDNA and not of genomic DNA. The sequences have been published [19]. Real time PCRs were performed in an ABI PRISM 7700 Sequence Detector (Applied Biosystems) following the standard procedure. The specificity of the PCR was confirmed by observing a single dissociation curve and no template controls were carried out to reject unintended amplification. Each sample was analyzed in duplicate obtaining an average Cq for sample. The relative amount of all mRNAs was calculated using the comparative 2^-ΔΔCq^ method and normalized to the reference *Rn18s* mRNA expression [17].

### Statistical analysis

The results are expressed as means ± SD. Comparisons were made using one-way ANOVA and the Tukey-Kramer multiple comparison test (post hoc) when the distribution of the variables was normal. When the variables did not show such a distribution (according to the Shapiro-Wilk test), or failed to show homology of variance, comparisons were calculated by the Mann–Whitney U-test. Correlations between variables were sought using the Pearson or Spearman correlation coefficients. SPSS version 15.0 (SPSS Inc, Chicago, IL) were used for calculations. Significance was set at P < 0.05.

## Results

### Effects of telmisartan or caloric restriction on body weight, plasma glucose and albuminuria

As shown in Table 1, mean body weight increased progressively during study period in all animals with a trend to a slower weight gain in the obese and diabetic Zücker rats when no treatment was administered (vehicle). At 19 weeks of age, mean body weight was only slightly greater in that vehicle group. The greater weight increase is statistically significant in the non-diabetic and the diabetic groups subjected to caloric restriction versus the vehicle group. Conversely we clearly see a marked increase in hyperglycaemia in that vehicle group over time, with statistically significant differences versus the other obese and diabetic animals that were subjected to any of the interventions.

**Table 1:** Follow-up of body weights and glucose concentrations

	**Body weight (g)**			**Glucose (mg/dL)**		
**Animal**	**10**	**19**	**Weight increase**	**10**	**19**	**Glucose increase**
Z	330 ± 23	517 ± 34	187 ± 35	150 ± 16	188 ± 78	38 ± 72
ZDFV	356 ± 22	433 ± 35^*^	76 ± 22^*^	201 ± 96	593 ± 63^*^	416 ± 103^*^
ZDFT	360 ± 14	480 ± 68	119 ± 62	223 ± 130	478 ± 115^*¶^	218 ± 121^*¶^
ZDFD	337 ± 13	495 ± 15^¶^	158 ± 14	146 ± 23	360 ± 119^*¶^	213 ± 130^*¶^

Differences between the experimental time-points (weeks 10 and 19) in the non-diabetic and diabetic obese rats. Data are shown as mean ± SD. Significant differences *p < 0.05 vs Zücker, ^¶^p < 0.05 vs ZDFV. The greater weight increase was seen in the non-diabetic rats (Z) and the fatty-diabetic animals subjected to caloric restriction (ZDFD). Conversely, in the vehicle group (ZDFV), we saw a trend to a slower weight gain but the most marked increase in hyperglycaemia over time.

Urinary excretion of albumin gradually increased in all groups as shown in Table 2. At 19 weeks, non-diabetic obese Zücker rats showed only a slight increase in urinary excretion, ranging from traces to microalbuminuria, whereas the urinary excretion of albumin in the vehicle group at the completion of the experiment was 10 times higher and only 3 to 4 times in the other two groups of diabetic rats (telmisartan and calorie-restricted diet). The increase in urinary albumin excretion was significantly higher in the vehicle group versus telmisartan and diet restricted groups. No statistically significant difference between caloric-restricted and telmisartan-treated animals was observed despite a trend towards a slower increase in the urinary albumin excretion in the drug group. Renal function and plasma creatinine values did not experience any significant change.

**Table 2:** Follow-up of plasma creatinine concentrations and urinary albumin excretions

**Animal**	**Serum creatinine (mg/dL)**			**Albuminuria (mg/day)**		
	**10**	**19**	**Creatinine increase**	**10**	**19**	**Albuminuria increase**
Z	0.25 ± 0.05	0.34 ± 0.11	0.05 ± 0.05	4.42 ± 0.86	37.20 ± 17.20	32.78 ± 17.52
ZDFV	0.45 ± 0.38	0.32 ± 0.10	-0.13 ± 0.33	57.31 ± 48.8	323.63 ± 134.3	260.37 ± 124.8*
ZDFT	0.40 ± 0.31	0.28 ± 0.12	-0.16 ± 0.21	24.73 ± 9.6	97.80 ±57.4^¶^	73.07 ± 54.2^¶^
ZDFD	0.20 ± 0	0.23 ± 0.05	0.03 ± 0.05	19.51 ± 7.81	130.32 ± 66.5^*¶^	107.62 ± 64.5^*¶^

Differences between the experimental time-points (weeks 10 and 19) in the non-diabetic and diabetic obese rats. Data are shown as mean ± SD. Significant differences *p < 0.05 vs Zücker, ^¶^p < 0.05 vs ZDFV. The urinary excretion of albumin in the vehicle group at the completion of the study period was significantly greater than in the other groups, either in the non-diabetic (Z) or in the fatty-diabetic animals with low-calorie diet (ZDFD) and telmisartan (ZDFT). Renal function and plasma creatinine values did not experience any significant change.

### Effects of telmisartan or caloric restriction on plasma lipids and lipoprotein

**Figure 1:**
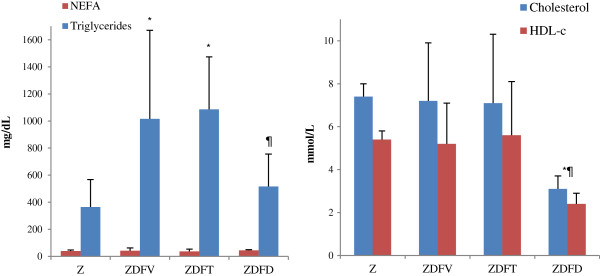
**Final values of plasma lipids concentration.** NEFA, Triglycerides, total Cholesterol and HDL-cholesterol. Differences between non-diabetic and diabetic obese rats at the end of experimental interventions. Data are shown as mean ± SD. Statistically significant differences *p < 0.05 vs Zücker ¶p < 0.05 vs ZDFV. Hyperlipidaemia in all diabetic groups (ZDF) was mainly due to elevated triglycerides with modest increases in total cholesterol, particularly HDL cholesterol levels, with the exception of the low-calorie diet group (ZDFD) in which cholesterol was significantly less than the others.

NEFA, triglycerides, total cholesterol and HDL-cholesterol values are summarized in Table 3. No statistically significant differences were found in circulating NEFA, whereas a marked and similar increase in plasma triglycerides was shown in the groups of obese and diabetic rats that there were not on a restricted diet regardless of they received vehicle or telmisartan treatment. Values in the non-diabetic and the diabetic with caloric restriction groups were only slightly different (lower in the first one), and both significantly different from drug-or-vehicle-treated animals (Figure 1).

**Table 3:** **Final values of plasma lipid, APOA1 and hepatic *Apoa1 *** **mRNA expression**

**Animal**	**NEFA (mg/dL)**	**Cholesterol (mmol/L)**	**HDL-c mmol/L**	**Triglycerides (mg/dL)**	**Plasma APOA1 (AU/L)**	**Hepatic *Apoa1 *** **(RAU)**
Z	39.1 ± 8.2	7.4 ± 0.6	5.4 ± 0.4	365 ± 202	0.64 ± 0.03	ND
ZDFV	42.4 ± 20.0	7.2 ± 2.7	5.2 ± 1.9	1016 ± 654*	0.90 ± 0.1*	1.0 ± 0.4
ZDFT	37.4 ± 16.7	7.1 ± 3.2	5.6 ± 2.5	1086 ± 388*	0.78 ± 0.1	0.75 ± 0.2
ZDFD	44.2 ± 6.1	3.1 ± 0.6^*¶^	2.4 ± 0.5^*¶^	516 ± 240^¶^	0.80 ± 0.06*	0.7 ± 0.3^¶^

Differences between non-diabetic and diabetic obese rats at the end of experimental interventions. Data are shown as mean ± SD. Statistically significant differences *p < 0.05 vs Zücker ^¶^p < 0.05 vs ZDFV. Hyperlipidaemia in all groups was mainly due to elevated triglycerides with modest increases in total cholesterol, particularly HDL cholesterol levels, with the exception of the low-calorie diet group (ZDFD) in which cholesterol was significantly less than the others. Fatty-diabetic rats showed higher APOA1 plasma concentration and hepatic mRNA expression than fatty-diabetic rats that were on low-calorie diet (ZDFD) or telmisartan treatment (ZDFT).

At the end of experimental intervention, hyperlipidaemia in all groups was mainly due to elevated triglycerides with modest increases in total cholesterol, particularly HDL cholesterol levels, with the exception of the low-calorie diet group in which total cholesterol and its HDL fraction were significantly less than a half than the others. Moderate correlation between serum triglyceride concentration and albumin urinary excretion at week 19 was also found (R = 0.530, p < 0.05).

### Effects of telmisartan or caloric restriction on plasma and hepatic expression of apolipoprotein A1

**Figure 2:**
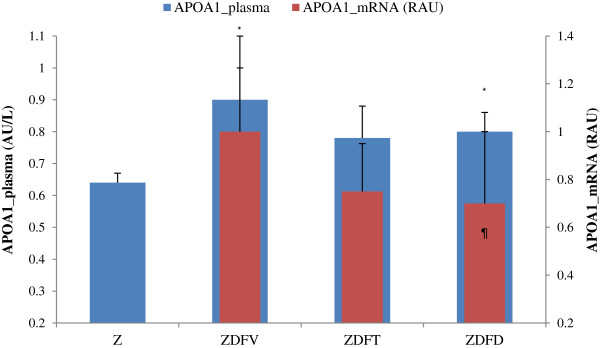
**Final values of plasma APOA1 and APOA1 hepatic mRNA.** Differences between non-diabetic and diabetic obese rats at the end of experimental interventions. Data are shown as mean ± SD. Statistically significant differences *p < 0.05 vs Zücker ¶p < 0.05 vs ZDFV. Fatty-diabetic rats showed higher APOA1 plasma concentration and hepatic mRNA expression than fatty-diabetic rats that were on low-calorie diet (ZDFD) or telmisartan treatment (ZDFT).

Plasma APOA1 concentration and its mRNA hepatic expression are shown in Table 3. Plasma levels in the non-diabetic rats were significantly lower than the others regardless of cholesterol and HDL-cholesterol concentration. In contrast, the obese and diabetic rats showed a trend towards higher plasma concentration. Either in the telmisartan or in the low-calorie groups plasma APOA1 levels were lower than in the vehicle group (diet vs vehicle, p = 0.05). APOA1 hepatic mRNA expression showed statistically significant differences between the obese and diabetic animals that were on restricted diet compared to the vehicle ones, in which values were higher (Figure 2).

### Effects of telmisartan or caloric restriction on lipoprotein oxidation

LDL and LDL + HDL from each group were incubated and oxidation was tested with the DCF method [17]. Results are summarized in Table 4. ROS content in LDL was markedly lower in the diet restricted group, suggesting less oxidation of this lipoprotein, whereas higher signals were obtained from de non-diabetic and the diabetic with vehicle treatment obese groups. After incubating LDL of each group with its own HDL, very little fluorescent signal change was found for all groups with the exception of the obese and diabetic rats receiving vehicle, where the signal decreased substantially.

**Table 4:** ROS content in LDL and in LDL incubated in presence of HDL

		**Media**	**SD**
B		45	8
Z	LDL	63	10
	LD + HDL	61	9
ZDFV	LDL	63	10
	LD + HDL	45	7
ZDFT	LDL	46	8
	LD + HDL	43	7
ZDFD	LDL	29	4
	LD + HDL	31	4

Data are expressed as arbitrary fluorescence units and are mean ± SD of the groups. ROS content in LDL was markedly lower in the diet restricted group. Very little fluorescent signal change was found for all groups with the exception of the obese and diabetic rats receiving vehicle when incubating with HDL.

### Association among parameters

**Figure 3:**
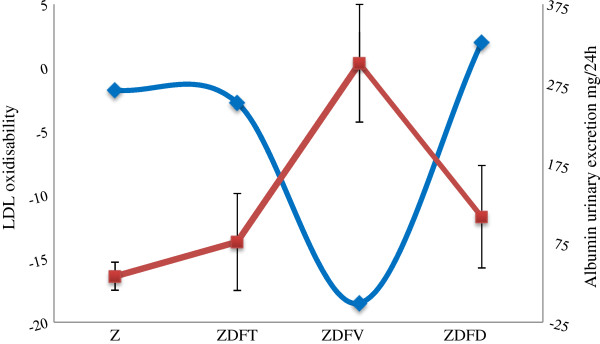
**Ability of HDL to inactivate ROS present in LDL and albuminuria in the different experimental groups.** Red line represents the increase in urinary albumin excretion between 10 and 19 weeks, and is expressed as mean ± SD in every group. Blue line represents LDL oxidisability as the difference between the average fluorescent signals on the DCF assay between the incubation with or without HDL of each group; the greatest DCF difference is seen in the vehicle group, with the largest amount of albuminuria.

At the end of the study in a bivariate analyses, we found significant correlations between the increase in the urinary albumin excretion and the glycaemia (R = 0.74, p < 0.05). APOA1 also showed a positive correlation with the urinary albumin excretion (R = 0.66, p < 0.05), while body weight increase was inversely correlated (R = - 0.73, p < 0.05). Results of the correlation between LDL oxidisability using DCF test with and without HDL, its average fluorescent signal of the difference, and media of urinary albumin excretion are depicted in Figure 3. Good correlations were also found between DCF signal difference between LDL and LDL + HDL and the increase in the urinary albumin excretion (R = 0.63) and inversely body weight increase (R = - 0.64).

## Discussion

The ZDF rat is an inbred rat model obtained through selective breeding (*fa* mutation) that results in shortened leptin receptor which does not effectively interact with leptin and is phenotypically expressed as obesity with high levels of leptin in the blood. When fed with a diet of Purina 5008, homozygote recessive males (*fa/fa*) develop obesity, hyperlipidemia, fasting hyperglycaemia and non-insulin dependent diabetes (NIDDM).

We observed a trend towards high glucose plasma concentration in all groups since the first time point. At week 10, hyperglycaemia was modestly higher in the vehicle group in which diabetes seems to develop earlier whereas low-calorie diet could delay the onset of a diabetic state similar to obese non-diabetic Zücker rats. Nine weeks later, the plasma glucose level in the vehicle group was up to three times higher than in the non-diabetic one, and almost two times than in the diet restricted group. This rapid increase in glucose level in the vehicle group due to an overt diabetic state was partially controlled by both interventions with telmisartan or low-calorie diet.

On the contrary, greater weight increase rate was found in the non-diabetic animals and minor in the diabetic vehicle group, whereas rats subjected to caloric restriction or telmisartan showed moderate increase during experiment. In work by Corsetti et al. as hyperglycaemia developed with a higher dietary fat-content weight increased slower [20]. Ohneda et al. also showed that when ZDF rats are fed with an unrestricted diet they became hyperglycaemic by 8 weeks of age, whereas all diet-matched rats remained euglycaemic despite the fact that at 18 weeks of age their mean body weight equalled that of obese rats on an unrestricted diet [21]. As we could see, rats on a moderate caloric restriction diet with a well-adjusted nutrient content showed a normal growth whereas animals on an unrestricted diet had only a markedly increase in body fat content but weight increase was hindered by the severe catabolic state associated with earlier and more severe diabetes as it was suggested by Corsetti and colleagues.

Telmisartan can modulate PPARγ by improving insulin sensitivity without fluid retention [14]. Upton et al. demonstrated that ZDF rats treated with experimental thiazolidinedione MCC-555 improved metabolic status and insulin sensitivity, and had a small but significant increase in body weight gain during experimental period whereas vehicle-treated animals did not [22]. Despite the marked hyperphagia in diabetic ZDF rats, they do not continue to gain weight after 15–20 weeks of age due to heavy losses of energy as glycosuria and higher catabolism. The body weight gain in diabetic ZDF rats following telmisartan treatment is probably the result of increase glucose entry into adipose tissue and the conversion into triglycerides.

Urinary loss of albumin in the vehicle treated diabetic group was significantly higher than groups on caloric restricted diet or telmisartan treatment at week 19, but also since week 14 (data not shown), as well as the increase in albuminuria since the starting point at the experimental period. Albuminuria correlates with endothelial dysfunction and predicts cardiovascular outcomes by its predisposition to systemic atherosclerosis [23].

With increasing age, fa/fa-rats spontaneously develop proteinuria and focal segmental glomerulosclerosis (FSGS), eventually leading to renal failure [24]. The development of proteinuria and/or FSGS in fa/fa-rats can be ameliorated by treatment with a 3-hydroxy-3-methyl-glutaryl-coenzyme A (HMG-CoA) reductase inhibitor [25]. When studying diabetic ZDF, Coimbra et al. showed that the animals exhibited pronounced hyperinsulinemia and hyperlipidaemia at 6 weeks and became diabetic after 14 weeks of age. Significant FSGS nephropathy was first noted in 18-week-old fa/fa-rats and tubulointerstitial damage and proteinuria in 40-week-old fa/fa-rats but progressive glomerular hypertrophy was already detected in diabetic fa/fa rats after 10. They suggested that early progressive podocyte damage and macrophage infiltration was associated with type II diabetes mellitus but also with hyperlipidaemia [26]. Noda et al. [27] examined the role of angiotensin II in obese Zücker rats and suggested that angiotensin II is the main cause of diabetic nephropathy as is the case in diabetic patients. Increase in proteinuria and albuminuria are inhibited by ACE-I or ARB in animals [28]. Therefore, as in the work by Ohmura et al. [29], it is possible that the renoprotective effect of telmisartan may be induced at least in part by inhibition of the renal hypertensive effect of angiotensin II based on the antagonistic activity to the AT1 receptor, but also by its non-hemodynamic effects in relation to cell proliferation, renal sclerosis and fibrosis stimulated by angiotensin II [30]. They showed that even at low doses, telmisartan inhibited the increase in albuminuria and was associated with a significant decrease in the progression of glomerulosclerosis. Our findings are consistent with their conclusions regarding those renal changes.

Our results suggest that a low-calorie diet can prevent urinary loss of albumin and therefore early renal damage probably by its lipid lowering effect and slowing the rapid decline in insulin sensitivity. Works by Sparks- Corsetti et al. showed a significant increase over time in serum cholesterol concentration in the higher dietary fat diet relative to the lowest fat-content diet and triglycerides concentration in male ZDF rats. Previously that group had reported an extensive characterization of the diabetic dyslipidaemia in obese ZDF rats that was initially due to triglyceride and free fatty acids but followed by an additionally rise in serum total cholesterol (LDL and HDL cholesterol) in the insulinopenic state, by 20 weeks of age [20,31]. In this study, telmisartan did not show the same effect as with restricted diet, probably due to low dose of treatment and short experimental period. According to Ohmura et al. telmisartan did only inhibit the increase in blood cholesterol and triglycerides with higher doses of telmisartan and longer treatment periods [26].

We also found moderate correlation between serum triglyceride concentration and albumin urinary excretion. As mentioned above, treatment of hyperlipidaemia can reduce glomerular injury in obese Zücker rats. Work by Joles et al. [32] showed that analbuminemic rats with hypertriglyceridemia developed podocyte injury and glomerulosclerosis, and that hypertriglyceridemia, proteinuria, and the increase in desmin staining (podocyte injury) were largely prevented by ovariectomy.

Regarding APOA1 either in serum or in hepatic tissue, to our knowledge, this is the first report on plasma APOA1 concentration and hepatic mRNA expression in ZDF rats subjected to low-calorie diet or telmisartan. Current data shows a trend towards higher plasma APOA1 concentration as well as mRNA hepatic expression when animals are not treated with telmisartan and are fed a normal chow. A study by Sparks et al. [31] in hyperinsulinemic and insulinopenic Zücker diabetic fatty rats showed increases both APOA1 and APOA4 levels more than doubled between 10 and 20 weeks in ZDF rats as hyperglycaemia and hypercholesterolemia developed. In our experiment conditions, the effect of PPAR modulation with low dose telmisartan and caloric restriction seems to be independent of serum cholesterol levels since there were no differences between vehicle and telmisartan-treated group. This could be explained by the non-linear relationship between HDL and APOA1 levels depending on the type of HDL-particle analysed [33]. A good correlation between serum APOA1 levels and the increase in glycaemia was observed in the present study. Murao et al. [34] showed that transcriptional activity of the rat *APOA1* promoter in HepG2 cells paralleled the endogenous mRNA expression, and this activity was dependent on the dose of glucose or insulin: glucose decreases and insulin increases *APOA1* promoter activity, respectively. We found that endogenous mRNA expression paralleled serum apolipoprotein concentration. However, animals with marked hyperglycaemia and severe diabetes showed higher serum APOA1 concentration suggesting a positive regulation *in vivo*.

Oxidation of LDL is thought to contribute to the development of atherosclerosis. Using the dichlorofluorescein analysis (DCF), the high fluorescent signal generated by LDL-oxidized-phospholipids in the vehicle group suggests a greater pro-inflammatory and pro-atherosclerosis state in the obese and diabetic animal group than the others. On the contrary, LDL from calorie-restricted diet resulted in low fluorescent signal. Navab et al. demonstrated that normal HDL inhibited the fluorescent signal generated by oxidized phospholipids in the cell-free assay using dichlorofluorescein, whereas patient (with definite coronary atherosclerosis) HDL did not. In our study when incubating this oxidized LDL from vehicle group with its own HDL, we saw a decrease in the fluorescent signal, suggesting intact beneficial properties of HDL in this early setting when preventing the formation or inactivating oxidized phospholipids. As mentioned above good correlations were also found between the DCF signal difference between LDL and LDL + HDL incubation and the increase in the urinary albumin excretion, suggesting that either LDL oxidisability or the properties of HDL in this animal model of type II diabetes could be used as marker of albuminuria and endothelial dysfunction. The low calorie diet lowered total serum cholesterol, although no dietary manipulations were made in fatty acid content that could increase the LDL oxidative resistance. The relative content in monounsaturated (MUFA) and polyunsaturated fatty acids (PUFA) were the same in both the normal chow and the 30%-less calorie diet. Nevertheless, we have clearly shown an inhibition of oxidative LDL signal in the restrictive dietary group compared with the others. This would be in agreement with the results of Hargrove et al. [35] who showed differences in LDL oxidative susceptibility between different diets (although the objective of the study was to evaluate the effects of diets high in MUFA from different food sources on LDL oxidative susceptibility). They compared these effects with those of a Step II blood cholesterol-lowering diet (25% total fat, 7% saturated) as well as a higher fat average American diet (35% total fat, 15% saturated). As regard to the two last diets, they showed that the average American diet tended to cause the shortest to LDL-oxidation lag time compared with the Step II diet.

Telmisartan may mediate cholesterol efflux by activating PPAR. In this regard, Matsumura et al. [36] demonstrated that telmisartan increased the expression of CD36, ABCA1 and ABCG1, all PPAR-responsive genes, and the activation of LXR, a transcription factor that also regulates ABCA1 and ABCG1 gene transcription in macrophages. This finding corroborated previous reports that telmisartan activated PPARγ target genes including CD36 in monocytes [37] and that telmisartan increased cholesterol efflux via PPARγ-dependent ABCA1 and ABCG1 expression in macrophages [38].

## Conclusions

In conclusion, this is the first study in which LDL oxidative susceptibility and response to HDL incubation, serum APOA1 as well as mRNA hepatic expression and urinary albumin excretion are analysed in an obesity related type II diabetes rat model subjected to either a dietary intervention or a pharmacologic modulation of PPAR gamma with telmisartan. Our results suggest important beneficial effects of both interventions in terms of LDL oxidative susceptibility and progression of albuminuria in obesity related type II diabetes.

## Abbreviations

ABCA 1: ATP binding cassette transporter A1; ABCG 1: ATP binding cassette transporter G1; ACE-I: Angiotensin II converting enzyme inhibitor; APOA1: Apolipoprotein A1; ARB: Angiotensin receptor blocker; AT-1: Angiotensin 1; DCF: Dichlorofluorescein; GLUT-1: Glucose transporter 1; FSGS: Focal segmental glomerulosclerosis; HDL: High density lipoprotein; LDL: Low density lipoprotein; LXR: Liver X receptor; MUFA: Monounsaturated fatty acids; NIDM: Non-insulin-dependent diabetes mellitus; PPAR: Peroxisome proliferator-activated receptor; PUFA: Polyunsaturated fatty acids; mRNA: Messenger ribonucleic acid; TG: Triglycerides; Z: Zücker; ZDF: Zücker diabetic fatty.

## Competing interests

No competing financial or non-financial interests exist.

## Authors’ contributions

AML participated in the animal handling and biochemical laboratory assays. CB was involved in the plasma lipid and apolipoprotein assays, prepared RNA and quantified mRNA. EJL participated in the analysis and interpretation of the data, the statistical analysis and preparation of the manuscript. IG was involved in the conception and design of the study. JO contributed to the design of the study, supervised the lipid and apolipoprotein assays and preparation of the manuscript. PI was involved in the conception and design of the study and participated in the animal manipulation and biochemical laboratory assays. All authors read and approved the final manuscript.
